# Traffic Sign Recognition Using Multi-Task Deep Learning for Self-Driving Vehicles

**DOI:** 10.3390/s24113282

**Published:** 2024-05-21

**Authors:** Khaldaa Alawaji, Ramdane Hedjar, Mansour Zuair

**Affiliations:** Computer Engineering Department, College of Computer and Information Sciences, King Saud University, Riyadh 11543, Saudi Arabia; zuair@ksu.edu.sa

**Keywords:** multi-task learning, deep learning, traffic signs, YOLOv7, self-driving vehicles, decision-making

## Abstract

Over the coming years, the advancement of driverless transport systems for people and goods that are designed to be used on fixed routes will revolutionize the transportation system. Therefore, for a safe transportation system, detecting and recognizing traffic signals based on computer vision has become increasingly important. Deep learning approaches, particularly convolutional neural networks, have shown exceptional performance in various computer vision applications. The goal of this research is to precisely detect and recognize traffic signs that are present on the streets using computer vision and deep learning techniques. Previous work has focused on symbol-based traffic signals, where popular single-task learning models have been trained and tested. Therefore, several comparisons have been conducted to select accurate single-task learning models. For further improvement, these models are employed in a multi-task learning approach. Indeed, multi-task learning algorithms are built by sharing the convolutional layer parameters between the different tasks. Hence, for the multi-task learning approach, different experiments have been carried out using pre-trained architectures like, for instance, InceptionResNetV2 and DenseNet201. A range of traffic signs and traffic lights are employed to validate the designed model. An accuracy of 99.07% is achieved when the entire network has been trained. To further enhance the accuracy of the model for traffic signs obtained from the street, a region of interest module is added to the multi-task learning module to accurately extract the traffic signs available in the image. To check the effectiveness of the adopted methodology, the designed model has been successfully tested in real-time on a few Riyadh highways.

## 1. Introduction

As part of the development of autonomous transportation systems, particularly driving assistance systems, several manufacturers and laboratories have oriented their work towards the exploitation of visual information due to its vital role in the detection of roads, vehicles, pedestrians, and traffic signs. Using a camera as an onboard sensor has the problem of external light intensity, further complicated as traffic signs are usually located in a small portion of the acquired image in an outdoor environment with variable weather, which increases the complexity of detection. Therefore, serious accidents may happen when drivers miss traffic signs due to a complex detection environment [1]. Therefore, it is imperative to overcome these barriers and accurately detect traffic signs in difficult situations. As a result, this improvement will reduce road accidents and road congestion.

Traffic sign (TS) recognition is the main issue for a driver assistance system as it has a dual role in warning and guiding the driver. However, detection and recognition of such signs is indeed challenging as their appearances are not consistent between countries. To overcome such challenges, existing methods could be modified with proper feature extraction and classifiers created from scratch to handle all sign categories. However, this solution would be a time-consuming task.

Recent advances in deep learning have shown promising results in the detection and recognition of general objects. Using a deep neural network model to extract the effective features from a road image is more effective than the conventional traffic sign recognition (TSR) algorithms. Such a neural network model simulates the structure of the human brain during the processing of information, which has the potential to improve the robustness and generalization of the algorithms [2,3]. In deep-learning models, millions of data instances are usually needed to learn accurate parameters. However, such requirements for high-size datasets cannot always be satisfied in practical applications. In such circumstances, multi-task learning (MTL) has proved to be an efficient recipe for exploiting useful information from other learning tasks that are related to the same problem, therefore alleviating this data sparsity problem. In an MTL model, machine learning paradigms are trained jointly with data acquired from multiple tasks, applying shared representations. Such representations boost the prediction performance and can probably improve the active learning speed. Furthermore, they help to lessen the recognized deficiencies of deep learning, namely the large-scale data requirements and computations [4]. In the domain of autonomous transportation systems, automated recognition of traffic signs has emerged to be a fertile research problem. Such systems have to be fast and efficient enough to detect traffic signs in real-time contexts and identify these signs precisely.

This paper is mainly focused on applying deep learning models with multi-task learning (MTL) to enhance the performance of autonomous navigation systems by increasing the recognition rate of traffic signs without country restrictions. To this end, an adequate dataset has been chosen (the most popular traffic signs). Indeed, traffic signs on the road are often blurry with low illuminance, which increases the complexity of the detection and classification of the autonomous driving system. To this end, the methodology followed to achieve the goal consisted of numerous steps, which are summarized below.

1. Recognition of synthetic traffic signs: select the most accurate deep neural network architectures from the single-task learning processes.
2. Use these CNN models in MTL (soft and hard parameter sharing structures) with different structures of learning (fine-tuning approach or training the entire network).
3. In the real world, the TSR needs to locate the traffic sign on a picture taken from the street. YOLOv7 was chosen and added to the architecture with the highest accuracy (MTL-SS).
4. Add a decision maker that explains the predicted traffic sign, which will be mapped to the action to be taken by the agent.

Therefore, the key contributions of this paper can be highlighted as follows:

A new deep learning model based on a convolutional neural network (CNN) combined with a YOLOv7 module is developed based on multi-task learning to improve the recognition rate for the traffic signs acquired by an autonomous system from the street.

## 2. Related Work

In the context of self-driving vehicles, various studies have been reported in the literature to investigate traffic sign detection and recognition problems using different approaches. In this section, works related to TSR based on image processing and machine learning are first addressed. After, the MTL architectures related to TSR are discussed.

### 2.1. Traditional Methods Based on Computer Vision and Machine Learning

Before the development of deep neural networks, traffic sign recognition focused on approaches based on several feature extraction methods combined with machine learning algorithms for classification purposes. The authors in [5] provided a critical review of traffic sign detections using computer vision segmentation and feature extraction. Although the authors in [6] presented a fairly thorough comparison of different segmentation approaches, this work covered only the use of segmentation. A dynamic pixel aggregation technique using the hue, saturation, and value (HSV) color space was proposed by the authors in [7]. The authors in [8] proposed a system that performs segmentation of chromatic and achromatic scene elements combined with a machine learning algorithm (SVM) and sign-shape classification using Fourier descriptors. The authors in [9] proposed to extract a red bitmap from the acquired image to detect the circular shape of traffic signs. Experimental results show that the method is efficient even in bad lighting conditions. However, this method is efficient only for circle-shaped traffic signs. The histogram of oriented gradients (HOG) is considered the most widely used feature extraction. The authors in [10] suggested a model based on traffic sign extraction built on a color probability model and a color HOG. The classification is performed using a CNN classifier. The random forest classification algorithm has been also used in [11], while in [12], the authors used shape and pictogram classification using support vector machines.

In recent years, the performance of traffic sign recognition has been increased by the introduction of deep machine learning algorithms. A comprehensive review of the methods using traditional machine learning and based on deep learning was provided in [13]. The authors in [14] implemented a detector by adopting the faster R-convolutional neural network (R-CNN) framework combined with the MobileNet architecture. The goal of the research achieved in [15] was a lightweight CNN to permit easy implementation, and the improved network LeNet-5 model was chosen for the classification of road signs. The authors in [16] raised the shortcoming of TSR methods based on using an end-to-end CNN. So, they proposed a finely crafted feature based on the color-histogram-based features and HOG features. The dimension of the extracted features is reduced by PCA, which reduces the running time of traffic sign recognition significantly.

### 2.2. Traffic Sign Recognition Based on Multi-Task Learning Approaches

Deep neural networks have been used for traffic sign recognition and have obtained promising results. However, most of the previous work is confined to one specific task of recognition, which restricts the achievement of high performance due to many parameters (like, for instance, a reduced dataset size). Therefore, the objective of multi-task learning [4] is to increase the generalization performance of all tasks, achieved individually, by utilizing valuable information available in numerous related tasks. To this end, the authors in [17] proposed a fast and accurate multi-task learning-based architecture for joint segmentation of drivable areas, lane lines, and classification of the scene. On the other hand, the authors in [18] applied a multi-output DNN model for simultaneously learning multi-task traffic classifications. The model is combined with a one-shot learning process. A new data-driven system to recognize all categories of traffic signs was proposed in [19]. The system includes both symbol-based and text-based signs captured from video sequences. The module contains three sub-systems. Detection occurs using traffic sign regions of interest. Then, a multi-task convolutional neural network is trained with a large amount of data for accurate classification and recognition. The third sub-system includes a post-processing module to make a recognition decision.

## 3. Multi-Task Deep Learning for Synthetic Traffic Sign Recognition

Rather than single-task learning, a new model is proposed in this study based on a multi-task learning approach. In this section, the multi-task learning approach and its advantages as compared to single-task learning are first presented. The concept of soft and hard parameter sharing in MTL is also discussed. Then, the proposed traffic sign recognition system is presented and discussed.

### 3.1. Multi-Task Learning Approach

**Figure 1:**
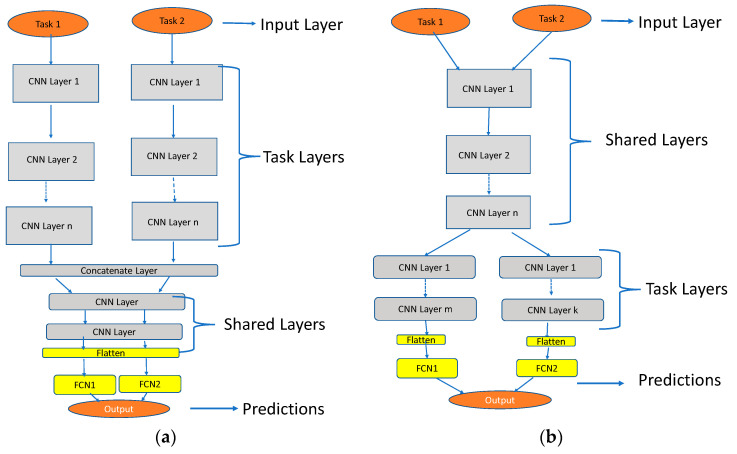
(**a**) MTL Soft Sharing (MTL-SS); (**b**) MTL Hard Sharing (MTL-HS).

Multi-task learning (MTL) is an efficient approach to train models using data gathered simultaneously from multiple tasks [20]. Shared representations are used to obtain common concepts and ideas from relevant tasks and consequently enhance accuracy and performance. They lead to faster learning as compared to a single task. Moreover, they help to mitigate the need for a large amount of data. In deep learning, MTL focuses on learning representations through either hard or soft parameter sharing of hidden layers of neural networks between all tasks [20]. Hard parameter sharing, shown in Figure 1b, is the most often used MTL technique. It is about sharing the hidden layers across all tasks while maintaining numerous task-specific output layers. The shared representations will help the model to reduce overfitting and increase the data efficiency. In contrast, a model using soft sharing has individual sets of parameters for each task, as shown in Figure 1a. Note that this work focused on this architecture.

Consequently, in this paper, the MTL approach is applied for a self-driving automated system to recognize a traffic sign. The MTL model is based on deep neural network architecture (CNN) to accurately extract the features from synthetic traffic sign frames. The model is trained jointly using two different datasets (traffic signs and traffic lights). Therefore, the MTL model accepts a synthetic sign image as input for both datasets and produces object labels as output.

**Figure 2:**
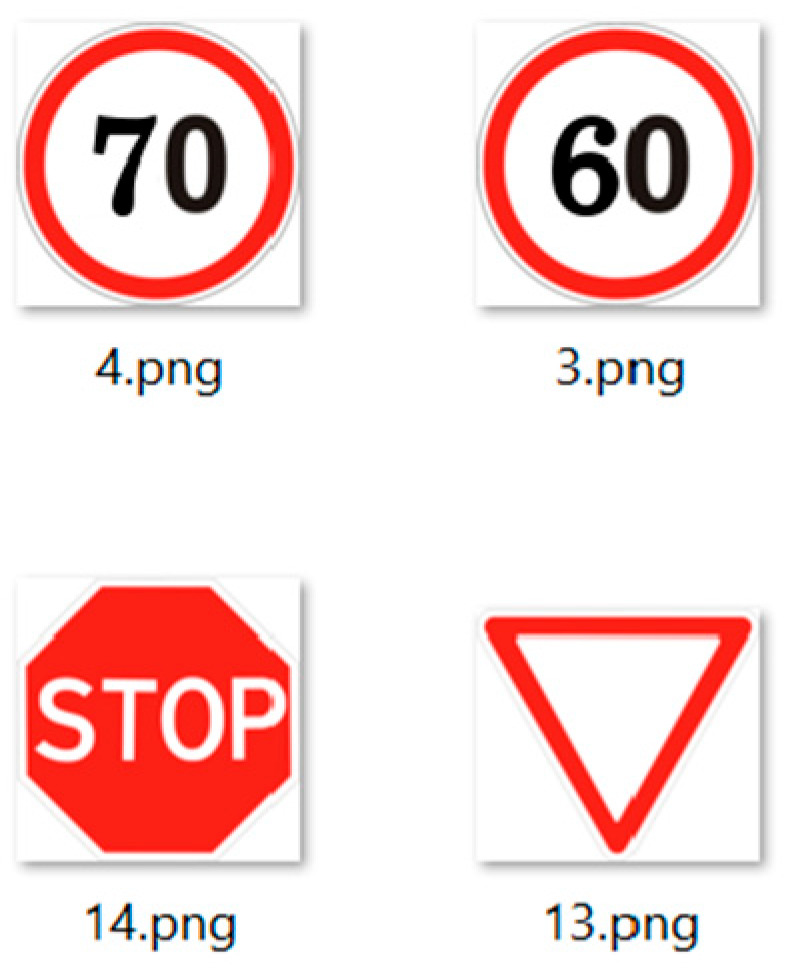
Samples of synthetic traffic signs [21,22].

Note that a sample of synthetic traffic signs that are sharp and clear is shown in Figure 2.

To choose the best architecture, firstly, multiple models using single-task learning were investigated and trained on the traffic signs and traffic lights datasets. Afterward, the optimal architecture was used in the MTL approach. Subsequently, a series of steps were performed to obtain the final output of the model and identify the type of traffic signal. Thus, the methodology to achieve the main goal of this research paper is to develop an accurate recognition system with an optimal decision for the agent.

### 3.2. Proposed Model

**Figure 3:**
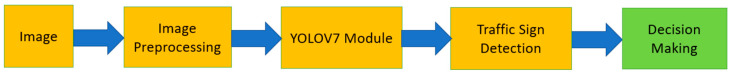
The proposed model.

The overall proposed model is illustrated in Figure 3. In the first step, several preprocessing techniques such as data augmentation are applied to the input images. Using the YOLOv7 model, the region of interest areas are extracted from each image, which contain the coordinates of the road signs. After that, an MTL-CNN model is built to classify the signs. Finally, the optimal decision is taken to assist the driving process of the vehicle.

**Figure 4:**
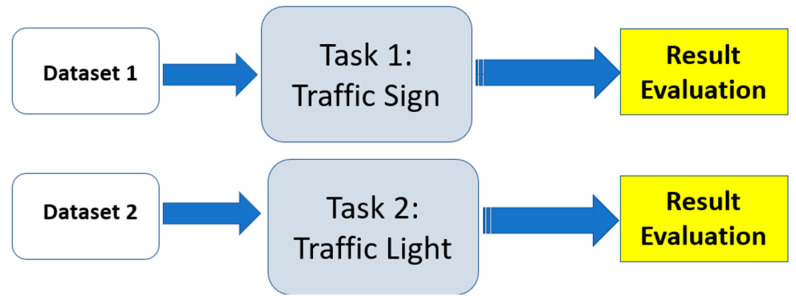
Evaluation of different classification models using single-task learning.

**Figure 5:**
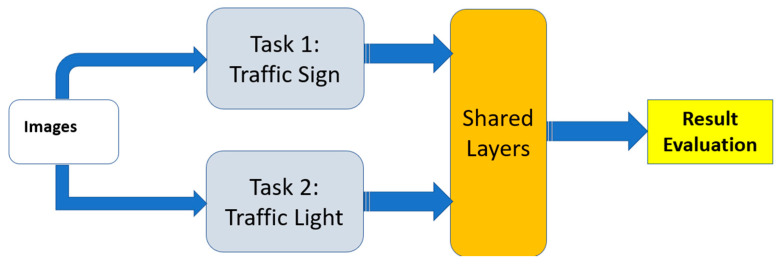
Using PCNNs for MTL-SS.

Note that for the classification block, numerous experiments are carried out utilizing the various architectures shown in Figure 1 to attain the designated aim starting from STL to MTL (MTL-HS and MTL-SS). The steps are briefly illustrated as follows:

1. It is known that multi-task learning can benefit from transfer learning (with a pre-trained model), where knowledge learned from one task is transferred to another related task. To this end, the well-known architectures have been trained independently using STL. Figure 4 shows the resultant pre-trained models that will be used in MTL. The details of this part are given in [23].
2. For MTL-HS, a conventional hard weight-sharing approach is used, in which the task datasets are merged and a deep neural network is used without task layers. This architecture is named in this work as MTL-HS.
3. To create a soft-shared neural network architecture, previous pre-trained neural network (PCNN) models are used as task-specific layers. The output of these PCNNs is concatenated to a new CNN as a shared layer between these two tasks (see Figure 5).
4. A region of interest (ROI) module is added based on the CNN architecture to extract the location of the traffic signs and the synthetic traffic lights from the street view images.

### 3.3. Region of Interest (ROI) Module

**Figure 6:**
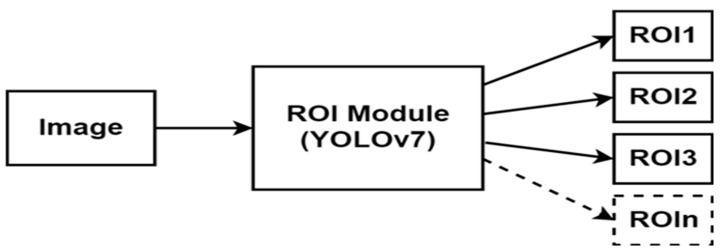
The ROI module is based on YOLOv7 using the LISA-GTSLDB dataset with the detection of different traffic signs and lights in a road image.

YOLOv7 (You Only Look Once version 7) is the latest official YOLO version which was released in July 2022 [24]. It is a fast and accurate real-time object detector that outperforms many object detectors such as YOLOR, YOLOX, ScaledYOLOv4, YOLOv5, DETR, Deformable DETR, DINO-5scale-R50, and ViT-Adapter-B in terms of speed and accuracy. It is used in many real-time applications such as self-driving cars, robotics, video analytics, and multi-object tracking. The author in [25] describes the structure of YOLOv7; it contains mainly the following parts: an input module, a backbone feature extraction network, feature pyramid networks, and a YOLO head classifier. This module was trained on the LISA-GTSLDB dataset from scratch to detect both traffic lights and traffic signs in real-time and extract the ROIs from the road image. The ROI module returns the X-coordinate of the top-left corner, the Y-coordinate of the top-left corner, the X-coordinate of the bottom-right corner, and the Y-coordinate of the bottom-right corner of the traffic sign bounding box, which defines the ROI of the image. As shown in Figure 6, the image is fed into the ROI module as an input, and the ROI module extracts the regions of interest from the image.

### 3.4. Decision-Making Module

The decision-making method is added to take the optimal decisions for each class. The decision can be viewed as the action to be taken by the agent. In this work, a Pandas data frame is used to correlate each sign with its decision. Essentially, it contains four columns: the ID of each class, the definition image, the label name, and the decision of each sign in the LISA-GTSLDB dataset. Such a data frame is used after extracting the ROIs from the image and making the prediction by MTL-CNNs. In this case, the correct decision will be made based on the class ID. For every prediction, there is a specific decision that is applied from the data frame. The data frame takes the output from the MTL model and makes a suitable decision for the self-driving car agent.

**Figure 7:**
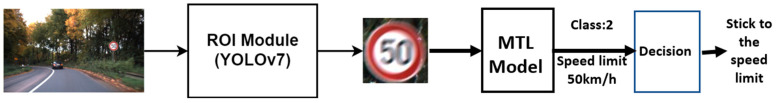
Different blocks of the system: the ROI module, the classification MTL model, and the decision model.

**Figure 8:**
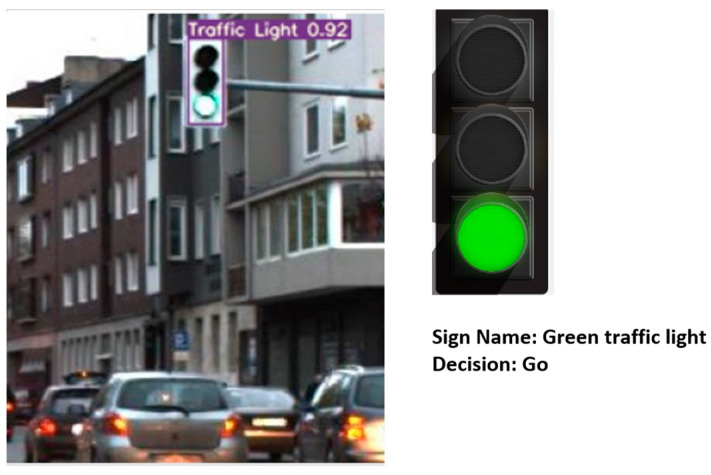
Real example of the entire system with the final output from the decision-making process.

Figure 7 shows the different blocks of the entire system. A full example of the process is illustrated in Figure 8. The ROI module extracts a synthetic traffic light as a region of interest, and the MTL model classifies it as a green traffic light; therefore, the final decision for the car is to go.

## 4. Experimental Results and Discussion

This section presents the results of the conducted experiments. First, the employed datasets in this study are briefly introduced. Then, the experimental results, particularly in the city of Riyadh, are presented. Scripts, based on Google Colab with Python 3.10, were created for training and testing stages. These scripts have been carried out on the 12th Gen Intel^®^ Core ™ i7-127000-2.10 GHz (Intel, Santa Clara, CA, USA) with NVIDIA GTX 1600 Ti (NVIDIA, Santa Clara, CA, USA).

### 4.1. Datasets

Three different datasets that are taken from the urban environment were employed to perform the experiments, composed of the German traffic sign recognition benchmark (GTSRB) [21], the German traffic sign detection benchmark (GTSDB) [22], and the LISA traffic light (LISATL) dataset [26]. These datasets were selected to carry out the experimental work. The datasets are unbalanced; therefore, data augmentation such as rotation, zooming, and cropping was performed using the augmenter package in Python to equalize the histograms of the datasets and improve the generalization capability before training the model [27].

GTSRB Dataset [21]: This dataset contains more than 50,000 images of traffic signs under 43 different classes with imbalanced class frequencies. In this study, the data is divided into 31,368 samples for training, 7841 samples for validation, and 12,630 samples for testing purposes.

GTSDB Dataset [22]: The GTSDB dataset is mainly used for image detection and image-based driver assistance applications. Each image in the dataset contains zero to six traffic signs. It is a successor to the GTSRB dataset. Like GTSRB, GTSDB contains more than 50.000 images with 43 different classes.

LISA Dataset [26]: This dataset contains more than 85.000 images of traffic lights under seven different classes (Go, Stop, Warning, Go forward, Go left, Warning left, Stop left) with imbalanced class frequencies. In this study, the data is also divided into 41,462 samples for training, 10,364 samples for validation, and 36,534 samples for testing purposes. Note that only three of the classes (Go, Stop, and Warning) were used for training and testing.

In the work presented in [23], the three datasets were used for single-task learning, as mentioned previously, to determine the best architecture to be used in MTL. In this work, these datasets were integrated as follows for multi-task learning:

LISA-GTSRB Dataset: It is worth mentioning that the GTSDB dataset does not contain any traffic light classes. Therefore, traffic light annotations have been added to the GTSDB by merging it with the LISA dataset. Thus, the final dataset is referred to as the LISA-GTSDB dataset.

LISA-GTSDB Dataset: This merged dataset contains 46 classes. Among the 46 classes, there are 3 classes for the first task and the remaining 43 classes for the second task. The first task is to detect traffic lights, while the second task is to detect traffic signs. Each class has a specific meaning and decision for the car. This process was performed by creating a CSV file that contains the important information for linking the sign signal with an appropriate decision.

Performance metric: In this work, the performance of different models is evaluated using testing samples from the dataset, and the accuracy, which is the overall percentage of correct predictions, of the overall testing samples is given by:

(1) $$Accuracy\left(ACC\right)=\frac{\left(TP+TN\right)}{\left(TP+FP+TN+FN\right)}$$

where TP stands for true positives, TN denotes true negatives, FP means false positives, and FN stands for false negatives. The Adam optimizer is used, with a learning rate of 0.0001 and a number of epochs of 20.

### 4.2. MTL Hard Sharing (MTL-HS)

In this subsection, different structures were tested for task layers in MTL-HS. Five types of pre-trained models, MobileNet, InceptionV3, DenseNet201, Xception, and InceptionResNetV2, were tested as a hard-shared layer on our merged dataset. Note that the pre-trained models reused in MTL-HS without task layers outperformed the architectures with specific task layers. CNNs and fully connected networks (FCNs) were added to these PCNNs for classification training purposes. Further, two types of loss functions are used: the standard loss function and the joint weighted loss function. The weighted loss function is given by Equation (2):

(2) $$L_{total}=\gamma L_{Task1}+\left(1-\gamma \right)L_{Task2}$$

where γ∈[0,1] is the weighting factor, and $L_{Task\;i}$ is the loss function of task *i.*

Once the models are set up, the supervised learning process is carried out using the following steps:

1. Importing the libraries
2. Importing the dataset.
3. Preprocessing and data augmentation,
4. Split dataset into training and testing parts.
5. Merge dataset into training and testing parts (LISA-GTSRB or LISA-GTSDB).
6. Specify the optimal hyperparameters.
7. Train the model (MTL-SS or MTL-HS) and evaluate the performance during the training process.
8. Plot the accuracy of the testing dataset.

As a result, it can be observed from Table 1 that the best pre-trained MTL-HS model with a standard loss function is Densenet201, with accuracy equal to 98.56%, while InceptionV3 achieves the best results when the gamma is equal to 0.5 (tasks are equally weighted), and DenseNet201 is superior when the gamma equal to 0.25 or 0.75, with accuracies of 97.86% and 98.16%, respectively.

### 4.3. MTL Soft Sharing (MTL-SS)

As was mentioned previously, many experiments have been conducted to determine the best architecture using MTL-SS. Note that only reasonable accuracies are shown in this work. Five experiments were performed using the pre-trained architectures InceptionResNetV2 and DenseNet201 since they produced the highest accuracies among other architectures during the single-task learning [23]. These pre-trained architectures were used at the task layer level. Table 2 describes the different changes incorporated into the different architectures used in single-task learning. The accuracy of the conducted experiments using soft sharing parameters is summarized in this table, where it can be observed that the highest accuracies of 98.96% and 99.07% were achieved under experiments 4 and 5, respectively.

### 4.4. Experimental Result in Riyadh

Real-time experiments were performed to demonstrate the efficacy of the developed model. In fact, images of Riyadh’s traffic signals were captured on a camera mounted on a car starting from the Qurtuba neighborhood and ending with Turki Al Awwal Street. Then, the images were fed into the system. A sample of the obtained experimental results is provided in Table 3, which highlights the effectiveness of the proposed solution. Some experiments were also conducted in the nighttime. Although the obtained results were satisfactory, it is worth mentioning that the results obtained during the day were more accurate than those acquired at nighttime.

## 5. Comparison with Existing Works

In comparison with relevant studies that exist in the literature, it can be observed that there are no studies that deal with the YOLO module, MTL, and decision-makers in a unified framework. For fair comparison, the proposed model is only compared with relevant works that employed MTL, as it is the main focus of this study. The comparison results are summarized in Table 4, wherein bold is used to represent the results of the proposed model.

## 6. Conclusions

Many countries plan to introduce self-driving vehicles to increase the safety of the transportation system and reduce road accidents. Traffic sign recognition is a major factor in road transportation safety. This work investigated traffic sign recognition as an important factor in self-driving vehicles. The main goal of this work is to further increase the recognition rate of traffic signs and lights of an autonomous system using images taken from the street with adverse weather conditions. Multi-task learning has advantages such as faster model convergence due to shared representation, which was used to recognize synthetic traffic signs. Since, on the road, synthetic traffic signs are difficult to locate, a recognition module based on YOLOV7 was added to the MTL module to locate and extract the desired traffic sign from the road images. Therefore, the multi-task approach was chosen for its benefits in improving prediction accuracy and increased data efficiency. The traffic sign (task 1) and traffic light (task 2) datasets were merged and used with several architectures based on deep learning approaches. It is notable that this work started with a single-task learning approach to choose an optimal model with high accuracy to be used in the MTL approach. Afterward, different architectures based on MTL wre built and experimented with evaluation using a testing dataset. Indeed, five different architectures were utilized, where the best achieved accuracy was about 98.96% (MTL-HS). In MTL-SS, the proposed model achieved a test accuracy of 99.07% when the entire network was trained. Based on the obtained results under the decision-making method, it also was observed that YOLOv7-ROI accurately located traffic signals and lights from images taken in the street. The entire developed system was effectively tested in real-time on the roads of Riyadh. Consequently, it is highly recommended to employ multi-task learning to effectively recognize street traffic signs, as it achieves the highest accuracy as compared to other approaches. Future work includes evaluating the proposed system on different datasets and analyzing its performance. Further, the drawback of the developed model in recognition of the traffic signs is its performance at night. This issue can be resolved by including detection algorithms for low-illumination and night scenes.

## Figures and Tables

**Table 1:** Test accuracy of MTL-HS on the merged dataset.

MTL-HS Architecture	Accuracy with Categorical Cross-Entropy Loss	Accuracy with Weighted Joint Loss		
		*γ* = 0.25	*γ* = 0.5	*γ* = 0.75
MobileNet	98.32%	97.36%	97.66%	96.45%
InceptionV3	97.65%	95.74%	97.81%	97.80%
DenseNet201	98.56%	97.86%	94.93%	98.16%
Xception	97.58%	97.67%	95.88%	97.35%
InceptionResNetV2	98.19%	96.36%	97.54%	95.15%

**Table 2:** Test accuracy of MTL-SS architectures.

Number	Architecture Model	Accuracy
1	Concatenated the output layers of the previous PCNNs (see Figure 1a for the architecture), added three classification layers (dense layers) with 1024 neurons each, and flattened the output layer with 46 neurons (Task 1 and 2). Note that the training was performed on the classification layers only.	98.05%
2	Used the same architecture except that only two classification layers with 1024 neurons were added with dropout layers to minimize overfitting in the network.	98.06%
3	Used only the extracted features of the two PCNNs (task layers) and added only one classification layer with 128 neurons and an output layer with 46 neurons.	97.91%
4	Used the same architecture as in experiment 3, but with an RMSprop optimizer to train the network.	98.96%
5	In the previous experiments, training focused only on the shared layers and classification layers. However, in this experiment, the entire network was trained (task layers and shared).	99.07%

**Table 3:** Experimental Result in Riyadh.

No.	Image from the Street	Output from the ROI Module	Sign Name	Decision
1	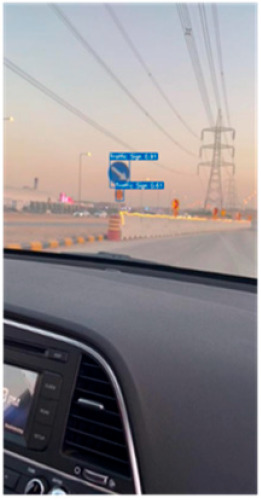	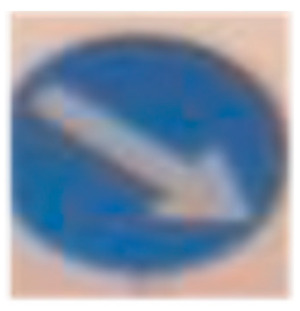	Keep right	Keep right
2	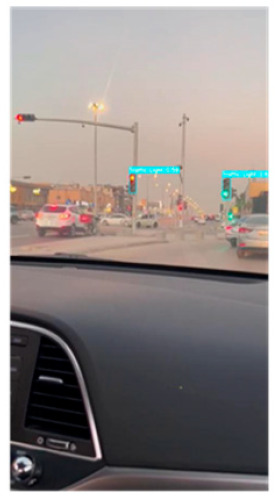	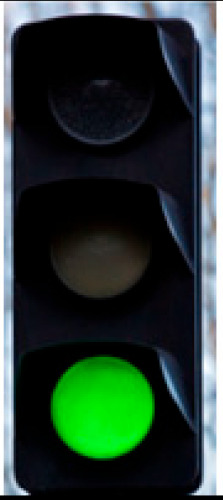	Green traffic light	Go
		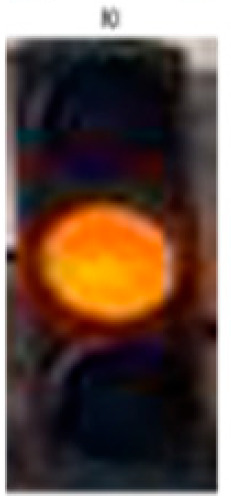	Yellow traffic light	Wait
3	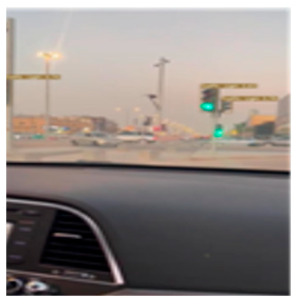	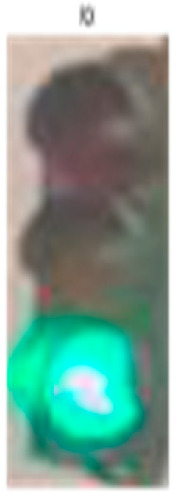	Green traffic light	Go
4	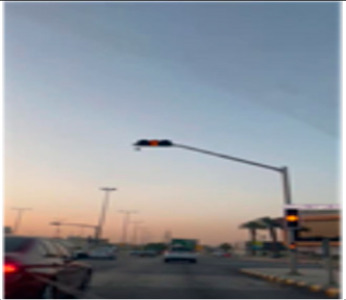	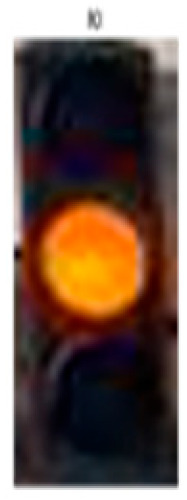	Yellow traffic light	Wait
5	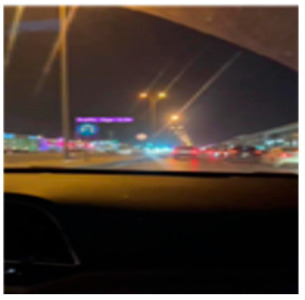	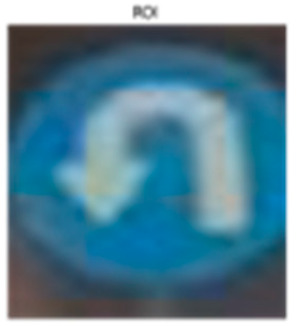	U-turn mandatory	All traffic must U-turn counterclockwise

**Table 4:** Comparison results with the existing literature (Bold represents the proposed models).

Model	Dataset	Accuracy
A shape symmetry detection algorithm [28]	GTSDB	93.96%
CNN [29]	GTSDB GTSRB	98.64%
R-CNN [17]	BDD	78.4%
A cascaded R-CNN [30]	GTSDB	98.7%
A cascaded R-CNN [30]	LISA	98.9%
MTL-SS (Training entire network)	LISA-GTSDB	**99.07%**
MTL-HS (DensNet201 as HS layer)	LISA-GTSRB	**98.56%**

## Data Availability

We used publicly available datasets.
